# Diphtheria—A Case

**Published:** 1892-08

**Authors:** L. Hoopes

**Affiliations:** West Chester, Pa.


### DIPHTHERIA—A CASE.

L. Hoopes, M. D., West Chester, Pa.

Miss R., aged about twenty, black hair, dark complexion, had diphtheria. Pain in all the limbs, back and head, high fever, nervous and restless, chills and heat alternate, fears she is taking typhoid fever. Sore throat commences on left side and went over to the right. Deposit upon and above both tonsils of a dirty yellow color; mucous membrane of fauces livid, and looked as if varnished; throat not sensitive to touch externally, complains of burning over whole body. Gave Lac-can$^{m}$, one dose in evening. Fever, nervousness, and pains disappeared within three hours and she slept well all night, which she had not done for three nights previously. Membrane all disappeared within thirty-six hours and steady improvement for three days, when returning soreness of left tonsil with sensitiveness to external pressure required a dose of Lach$^{2c}$, which relieved at once and the patient was discharged well.

